# From Synthesis to Application: Functionalized Magnetic Nanoparticles as a Simple and Reliable Tool for Nucleic Acid Purification

**DOI:** 10.1021/acsomega.5c06432

**Published:** 2026-01-21

**Authors:** Iuly Guimarães Ribeiro, Thais de Andrade Silva, Ana Carolina de Lima Barizão, Giordano Toscano Paganoto, Gabriel Fernandes Souza dos Santos, Sérvio Tulio Alves Cassini, Marco Cesar Cunegundes Guimarães, Jairo Pinto de Oliveira

**Affiliations:** † 28126 Federal University of Espírito Santo, Campus Maruípe, Av. Marechal Campos 1468, Vitória, Espírito Santo 29.040-090, Brazil; ‡ Center of Research, Innovation and Development, Laboratory of Environmental Characterization, Cariacica, Espírito Santo 29.140-130, Brazil

## Abstract

Magnetic nanoparticles (MNPs) are widely used for nucleic acid (NA) extraction, but their performance strongly depends on the synthesis and surface functionalization. In this work, we applied a design of experiments (DoE) approach to optimize the coprecipitation synthesis of iron oxide nanoparticles, identifying NH_4_OH flow rate and reaction temperature as the key factors. Under optimal conditions (5.5 mL min^–1^, 65 °C), Fe_3_O_4_ nanoparticles were coated with SiO_2_ and subsequently functionalized with (3-aminopropyl)­triethoxysilane (Fe_3_O_4_@SiO_2_-APTES). The resulting nanoparticles (∼12 nm) were stable and magnetically responsive and provided efficient NA binding. Their performance in NA extraction was validated by RT-qPCR, yielding Ct values (20–25 for S, ORF, and N genes) comparable to those of both silica column and commercial magnetic bead methods. These results demonstrate that DoE is an effective strategy for tailoring nanoparticle synthesis and highlight Fe_3_O_4_@SiO_2_-APTES as a simple, cost-effective, and reliable material for nucleic acid purification in routine laboratory applications.

## Graphical abstract

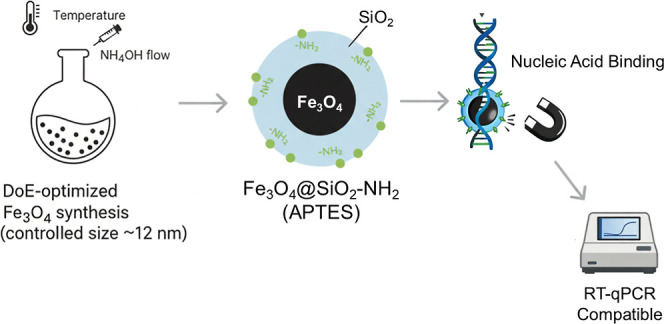

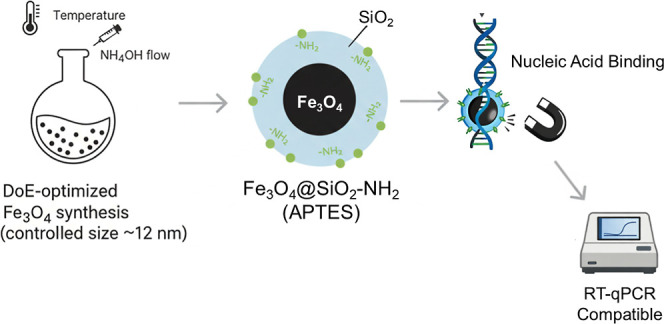

## 1 Introduction

The growing demand for rapid, sensitive, and high-throughput molecular diagnostics has intensified the need for efficient nucleic acid (NA) extraction methods.^1,2^ The purity and integrity of DNA or RNA are critical to the performance of downstream techniques such as polymerase chain reaction (PCR), next-generation sequencing (NGS), and nucleic acid–based biosensors.^3–6^ However, conventional extraction approaches, including phenol-chloroform and column-based protocols, are often time-consuming, involve toxic reagents, and offer limited compatibility with automated platforms.^7^

Recent advances in molecular diagnostics have underscored the importance of NA extraction platforms that are not only efficient but also reproducible, scalable, and compatible with downstream applications, such as PCR, NGS, and biosensing. Functional nanomaterials, particularly magnetic nanoparticles (MNPs), have emerged as enabling tools in this context because their tunable surfaces allow selective binding, rapid separation, and integration into automated diagnostic workflows.^8,9^ For instance, silica coatings not only provide colloidal stability and biocompatibility but also protect the iron oxide core from oxidation and agglomeration. In addition, the silanol groups on the SiO_2_ surface enable covalent coupling with silane agents such as APTES, which introduce outward-facing amino groups that strengthen electrostatic interactions with nucleic acids.^10^

Beyond their intrinsic magnetic properties, the surface engineering of MNPs plays a decisive role in diagnostic applicability.^11–13^ Recent studies have highlighted the versatile biomedical applications of MNP nanoparticles for targeted drug delivery, antimicrobial therapy, and bioimaging due to their excellent magnetic properties and biocompatibility.^14–16^ Despite extensive development, challenges remain in achieving cost-effective and widely accessible alternatives to commercial extraction kits, which often rely on complex chemistries, high reagent costs, or toxic solvents. In this regard, the optimization of coprecipitated Fe_3_O_4_ nanoparticles and their subsequent functionalization with TEOS and APTES represents a promising approach to balance synthetic simplicity, reproducibility, and performance. The SiO_2_ coating introduced via TEOS thus provides a stable and modifiable interface for APTES functionalization, ensuring both nanoparticle stability and effective nucleic acid capture. This strategy offers an application-driven pathway to develop functional nanomaterials that are not only structurally stable but also highly effective in recovering nucleic acids for diagnostic assays.

In response to these limitations, MPs have emerged as a promising alternative, enabling rapid, selective, and automatable separation of NA from complex samples.^17–19^ MP-based systems have gained significant attraction in commercial nucleic acid extraction kits due to their scalability, facile operation, and potential for integration into automated workflows. Most commercially available platforms rely on micrometer-sized magnetic beads functionalized with silica or carboxyl groups, which facilitate the adsorption of nucleic acids via electrostatic or chaotropic interactions under optimized binding conditions.^3,20–23^ Further, to enhance the extraction efficiency of NA, several types of MPs and their modifications have been explored for this purpose, each offering distinct advantages and efficiencies. While these systems generally provide satisfactory recovery rates, limitations related to their surface-to-volume ratio and magnetic responsiveness can impair binding efficiency and elution kinetics, particularly when processing low-concentration or degraded samples.^23,24^

To overcome these constraints, recent studies have focused on synthesizing magnetic nanoparticles (MNPs) with different properties, including particle size, surface charge, and coating composition, to enhance extraction performance. Functionalization with ligands such as amines, polyethylene glycol (PEG), or carbohydrate moieties, as well as the incorporation of nanostructured shells like silica or graphene oxide, has been shown to improve selectivity, reduce nonspecific binding, and increase stability across a range of pH and ionic strength conditions.^17–19,25,26^ These design strategies have significantly expanded the applicability of MNP-based platforms, enabling more robust, sensitive, and scalable workflows that meet the demands of modern molecular diagnostics.

Recent advances in material design have demonstrated that the physicochemical properties of MNP, particularly particle size, surface charge, and surface modifications, significantly influence NA extraction efficiency. For instance, Adams et al. (2015) demonstrated that surface coatings such as silica, oligo, and sequence-specific oligonucleotides significantly affect binding specificity and kinetics, with silica-coated beads effectively isolating total RNA, oligo beads selectively enriching mRNA, and sequence-specific beads enabling targeted capture of viral RNA and microRNAs.^27^ Moreover, Szymczyk et al. (2022) explored Fe_3_O_4_-based MNPs modified with various coatings, including polyethylenimine (PEI), gold, silica, and graphene oxide (GO) derivatives.^17^ They found that Fe_3_O_4_@PEI nanoparticles exhibit near-complete DNA binding through strong electrostatic interactions, while Fe_3_O_4_@GO–COOH coatings facilitate efficient DNA release via hydrophobic and hydrogen bonding mechanisms. Further, Ali et al. (2022) designed core–shell MNPs featuring cationic imidazolium-functionalized silica coatings coupled with triethylene glycol spacers, which synergistically enhance nucleic acid binding efficiency and biocompatibility through combined electrostatic and hydrogen bonding interactions.^24^ Complementing these functionalization strategies, Tjoa et al. developed TEOS-modified magnetic nanoparticles (TMNPs) with a crystalline size of 19.8 nm and ferromagnetic properties. The TMNPs demonstrated their effectiveness for DNA extraction from a wide range of bacterial species, including *E. coli*, *Salmonella* sp., and *M. tuberculosis*. By optimizing the binding buffer to 2.5 M guanidine thiocyanate (GuSCN) at pH 6.5, it achieved DNA yields ranging from 2.55 to 12.45 μg with 260/280 purity ratios between 1.58 and 2.27. The extracted DNA showed sufficient quality for downstream applications such as probe-based and intercalating dye-based qPCR, underscoring the critical role of buffer composition in maximizing the performance of functionalized MNPs for diagnostic workflows.^28^ Additionally, Prasetya et al. (2025) developed an environmentally friendly synthesis of magnetic-silica particles (MAGSi) by eliminating the washing step during Fe_3_O_4_ nanoparticle preparation, reducing waste and time. The unwashed MNPs (∼100 nm) were successfully silica-coated, forming larger MAGSi (∼1700 nm) with lower magnetization due to a thicker SiO_2_ layer. Despite this, they effectively extracted RNA and DNA from viral and bacterial samples, demonstrating strong performance in PCR and qRT-PCR assays.^29^ In this sense, these studies emphasize how advanced surface functionalization combined with process optimization drives the development of highly efficient and versatile MNP platforms tailored for nucleic acid extraction applications.

Despite the promising applications of MNPs, challenges persist in developing homogeneous and stable systems, particularly in controlling the interactions among nanoparticles and between nanoparticles and the carrier fluid.^30^ Given that magnetic properties are closely linked to particle composition and morphology, it is essential to carefully select synthetic methods that allow precise control over stability, size distribution, and crystallinity.^31–34^ Among the chemical methods available, coprecipitation is the most widely employed, relying on the simultaneous precipitation of Fe^2+^ and Fe^3+^ ions in an alkaline medium.^35–37^ However, despite its simplicity and scalability, this method often suffers from poor reproducibility and limited control over nanoparticle uniformity. In this work, we optimized Fe_3_O_4_ nanoparticle synthesis via response surface methodology and subsequently functionalized the particles with TEOS and APTES. These engineered nanoparticles were then evaluated for nucleic acid extraction performance, offering a reproducible, simple, and cost-effective alternative to conventional protocols and commercial magnetic bead-based kits.

## 2 Materials and Methods

### 2.1 Materials

Analytical-grade reagents were used in the synthesis of magnetic nanoparticles without further purification. The following chemicals were employed: ferric chloride hexahydrate (FeCl_3_·6H_2_O, Sigma-Aldrich, F2877), ferrous chloride tetrahydrate (FeCl_2_·4H_2_O, Sigma-Aldrich, 44939), ammonium hydroxide (NH_4_OH, Sigma-Aldrich), ultrapure water (Milli-Q, Synergy UV), absolute ethanol (VETEC 103), argon (OXIVIT, 99.99%), tetraethyl orthosilicate (TEOS, Sigma-Aldrich, 86578), and 3-aminopropyltriethoxysilane (APTES, Sigma-Aldrich, 440140). For comparison, nucleic acids were extracted with the Extracta Kit Fast DNA and RNA Viral (Loccus, São Paulo, Brazil), and PCR amplification was performed with the TaqPath COVID-19 CE-IVD RT-PCR Kit (Thermo Fisher Scientific, Waltham, MA, USA). All glassware was cleaned with a mixture of HNO_3_ and HCl (1:3), rinsed with distilled water, and washed with ultrapure water to eliminate possible contaminants.

### 2.2 Experimental Design

A systematic experimental design was employed to optimize the synthesis of magnetic nanoparticles, focusing on minimizing particle size distribution (PSD), which correlates with increased surface area and improved nucleic acid extraction efficiency.^34,38,39^ Initially, a literature survey was conducted to identify the primary variables affecting MNP synthesis, which included reaction time, temperature, Fe^2+^/Fe^3+^ molar ratio, stirring speed, and flow rate of ammonium hydroxide (NH_4_OH) (Table S1, Supporting Information). Based on this preliminary analysis, a fractional factorial design (2^5–1^) was employed to screen the significance of these five variables (Table 1). The analysis revealed that the temperature and NH_4_OH flow rate were the most influential parameters affecting particle formation and dispersion.

**Table 1:** Variables and Levels of the Fractional Design 2^(5–1)^

	levels	
variables	(−)	(+)
time (min)	10	180
temperature (°C)	25	100
molar ratio (FeCl_2_/FeCl_3_)	1	3
stirring speed (RPM)	200	600
NH_4_OH flow (mL min ^–1^)	1	10

Following the screening phase, temperature and NH_4_OH flow rate were identified as the most significant factors affecting PSD. These two variables were subsequently optimized using a face-centered central composite design (CCD), which allows the estimation of quadratic effects and facilitates response surface modeling (Table 2). All statistical modeling and analysis were performed using Statistica 12 (trial version), and model adequacy was evaluated through the analysis of variance (ANOVA), response surface plots, and residual analysis.

**Table 2:** Variables and Levels of the Fractional (2^3^)

	levels		
variables	(−)	(0)	(+)
temperature (°C)	30	65	100
NH_4_OH flow (mL min^–1^)	0.5	5.5	10.5

### 2.3 Synthesis and Functionalization of Nanoparticles

The synthesis of Fe_3_O_4_ nanoparticles was carried out under controlled conditions, in accordance with the experimental levels defined in the factorial design. Aqueous solutions of FeCl_2_·4H_2_O (0.03 mol·L^–1^ or 0.06 mol·L^–1^; 10 mL) and FeCl_3_·6H_2_O (0.03 mol·L^–1^; 10 mL) were mixed in a three-necked round-bottom flask fitted with a reflux condenser and maintained under continuous heating and stirring. The reaction was conducted under an inert nitrogen atmosphere to prevent premature oxidation of iron species.

Subsequently, 1.0 mL of NH_4_OH (28%) was added dropwise as a precipitating agent to initiate the coprecipitation of Fe^2+^ and Fe^3+^ ions, forming magnetite nanoparticles (Fe_3_O_4_). After synthesis, the particles were isolated and dried at 70 °C and then stored in Falcon tubes under dry conditions until further use in the functionalization steps.

### 2.4 Stabilization of Magnetic Nanoparticles

The magnetic nanoparticles (MNPs) were stabilized using a modified Stöber method, adapted from Thangaraj et al. (2019).^40^ Initially, 1.766 g of Fe_3_O_4_ nanoparticles were resuspended in 88 mL of a 10:1 ethanol-to-water solution and subjected to ultrasonic dispersion for 30 min to ensure uniform suspension. Following this, 0.35 mL of tetraethyl orthosilicate (TEOS) was added and mixed until homogeneity was achieved. To initiate the silica coating, 5.3 mL of NH_4_OH (28%) was added dropwise under constant stirring. The reaction mixture was maintained at 28 °C and stirred at 250 rpm for 20 h to allow complete hydrolysis and condensation of TEOS, resulting in silica-coated magnetic nanoparticles (Fe_3_O_4_@SiO_2_). The particles were then washed repeatedly with deionized water and ethanol until no detectable TEOS remained in the supernatant and dried at 70 °C.

For surface functionalization, 3.7626 g of Fe_3_O_4_@SiO_2_ were resuspended in a solution of 30 mL ethanol and 2.26 mL of (3-aminopropyl)­triethoxysilane (APTES). The mixture was sonicated for 30 min to promote interaction between APTES and the silica surface, followed by stirring at 180 rpm for 1 h at 30 °C. This procedure yielded APTES-functionalized nanoparticles (Fe_3_O_4_@SiO_2_–APTES), suitable for subsequent nucleic acid extraction assays. After functionalization, the nanoparticles were purified using a neodymium magnet placed against the centrifuge tube wall, which allowed for magnetic separation of the solid phase. The supernatant containing unreacted or excess APTES was carefully removed, and the particles were washed repeatedly to ensure removal of nonbound species.

The stability of APTES-coated magnetic nanoparticles was evaluated through UV/vis spectroscopy and Dynamic Light Scattering (DLS) and Zeta Potential (ZP) in different conditions of pH, saltiness, temperature, and shelf life.

### 2.5 Degree of Functionalization

The degree of functionalization with amino groups was estimated following the acid–based titration protocol proposed by Moaseri et al. (2013)^41^ with modifications. Briefly, 120 mg of Fe_3_O_4_@SiO_2_-APTES were added to 60 mL of HCl 0.02 M. The mixture was stirred for 30 min in a sealed container under a N_2_ atmosphere. After that, the supernatant was separated from magnetic nanoparticles and was titrated with NaOH (0.05 M) as the titrant.

The consumed HCl by Fe_3_O_4_@SiO_2_-APTES was accounted to be equivalent to the amount of amine functions present on the nanoparticle surface. Therefore, the difference between initial moles of HCl and the HCl consumed by NaOH represents the amount of amine functions on MNPs. Consequently, the degree of functionalization was calculated through eq 1

1 $$degree\;of\;functionalization=\frac{mmol\;of\;NH_{2}}{grams\;of\;MNP}=\frac{C_{A}\left(mL\right)\cdot V_{A}\left(M\right)-C_{B}\left(mL\right)\cdot V_{B}\left(M\right)}{sample\;weight\;\left(g\right)}$$

### 2.6 Characterization of the Nanoparticles

The synthesized Fe_3_O_4_@SiO_2_-APTES were comprehensively characterized to assess their morphology, size distribution, crystallinity, surface chemistry, and colloidal stability. Transmission Electron Microscopy (TEM) was employed to evaluate the morphology and particle size by using a JEM-1400 microscope (JEOL, USA Inc.), operated at 120 kV with a tungsten filament. The crystalline structure was analyzed via X-ray Diffraction (XRD) using a Philips PW 1710 diffractometer equipped with Cu Kα radiation. The diffraction patterns were recorded over a 2θ range of 30° to 90°, with a step size of 0.01° and a time constant of 2 s.

Optical properties were investigated by using UV–visible (UV–vis) spectroscopy in the 200–800 nm range, employing an Ocean Optics USB 2000 spectrophotometer. Raman spectroscopy was performed using a Metrohm Instant Raman Analyzer (MIRA DS), equipped with a 785 nm excitation source and a spectral range of 400 to 2300 cm^–1^. Fourier Transform Infrared (FTIR) spectra were acquired using an Agilent Cary 630 spectrometer to identify functional groups on the nanoparticle surfaces.

Hydrodynamic diameter and colloidal stability were determined via DLS and ZP measurements, respectively, using a Litesizer 500 (Anton Paar) instrument with 2 mL of colloidal suspension per measurement. DLS results were expressed in nanometers (nm), and ZP values in millivolts (mV). All characterization data were analyzed and processed using the OriginPro 8.5 SR1 software.

### 2.7 Extraction and Quantification of Nucleic Acids

This study evaluated the performance of Fe_3_O_4_@SiO_2_–APTES nanoparticles in extracting RNA from eight anonymized human saliva samples from individuals potentially infected with SARS-CoV-2. The project received approval from the institutional Ethics Committee (CEP), under protocol number 30993920.1.0000.5071.

RNA extraction using the synthesized nanoparticles was compared against two established protocols: manual extraction with a silica membrane column (Bio-Gene DNA/RNA Extraction Kit, Bioclin) and automated extraction with commercial magnetic beads. All reverse transcription-quantitative polymerase chain reaction (RT-qPCR) measurements were performed in triplicate for each sample and extraction method, minimizing intra-assay variability and ensuring reproducibility.^19^ In both protocols, the standard solid-phase material was replaced with the synthesized nanoparticles, while all other extraction conditions remained consistent.

The manual extraction process included cell lysis, nucleic acid binding, washing, and elution. During the binding step, the Fe_3_O_4_@SiO_2_–APTES nanoparticles served as the capture medium for RNA through magnetic separation. The same substitution occurred in the automated method, where the nanoparticles replaced commercial magnetic beads. A detailed description of the nucleic acid extraction protocols, including manual silica column-based and automated magnetic bead-based methods, is provided in the Supporting Information (Table S2).

RT-qPCR analysis was performed using a QuantStudio 5 system (Thermo Fisher Scientific, Waltham, MA, USA) with the TaqPath COVID-19 CE-IVD RT-PCR Kit, following the manufacturer’s protocol. The extraction solution used in these protocols had pH 6. The relative expression of viral gene markers was quantified using DataAssist software (Thermo Fisher Scientific), and the performance of the developed nanoparticles was compared to that of the two reference extraction methods.

## 3 Results and Discussion

### 3.1 Synthesis of Magnetic Nanoparticles and Factorial Experiments

The choice of iron salt significantly influences the synthesis, crystallization, and morphology of magnetic iron oxide nanoparticles. As discussed by Tanaka et al. (2022), the anionic component of the iron salt, such as sulfate, chloride, or nitrate, affects nucleation, particle growth, and the final nanoparticle structure. For example, sulfate ions strongly coordinate with Fe^3+^, which can inhibit certain iron oxide phases and lead to needle-like or nonmagnetic structures, whereas nitrate ions exhibit weaker coordination, resulting in less pronounced effects on particle formation.^42,43^

In our work, we selected iron chloride salts (FeCl_3_ and FeCl_2_) due to several advantages: the moderate coordination of chloride ions stabilizes Fe^3+^ in solution without strongly hindering nucleation or growth, enabling controlled formation of uniform nanoparticles; the high solubility of chlorides ensures a homogeneous reaction medium; and precipitation from chlorides generates minimal byproducts, preserving the magnetic properties of the resulting particles. Moreover, the FeCl_2_/FeCl_3_ coprecipitation method is a well-established and reproducible route that facilitates particle size and morphology control.^44,45^

Therefore, while the specific choice of iron salt can strongly impact the formation and characteristics of magnetic nanoparticles, the use of chloride salts in our study offered an optimal compromise between solubility, reaction control, and structural integrity, yielding Fe_3_O_4_ nanoparticles with high magnetic quality and suitability for nucleic acid extraction.

A preliminary review of the literature identified five synthesis parameters as potential influencers of MNP size: reaction time, temperature, Fe^2+^/Fe^3+^ molar ratio, NH_4_OH addition flow rate, and stirring speed (Table S1, Supporting Information). These variables were systematically evaluated using a 2^5–1^ fractional factorial design, with hydrodynamic diameter (nm), as determined by DLS, serving as the response variable (Tables S3 and S5, Supporting Information).

The Pareto chart from the fractional factorial design (Figure 1A) identified temperature as the only statistically significant variable influencing the nanoparticle size. However, because the CCD requires at least two variables for effective modeling, the NH_4_OH flow rate, ranked as the second most influential factor, was also included. This selection enabled a more comprehensive exploration of synthesis conditions and allowed for the assessment of potential interaction effects between temperature and NH_4_OH flow rate (Figure 1B,C; Tables S4 and S6, Supporting Information).

**Figure 1:**
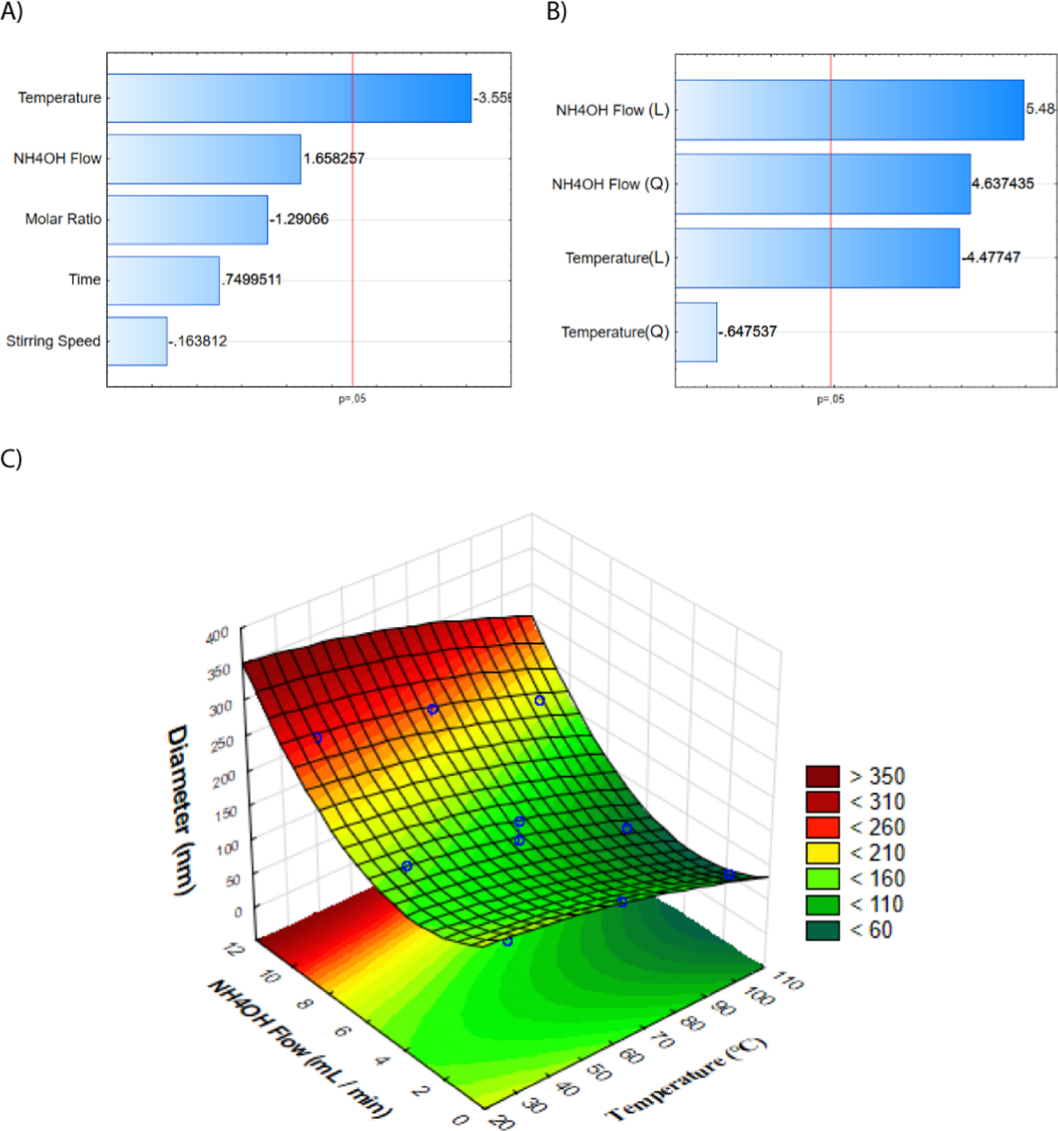
(A) Pareto chart from the 2^5–1^ fractional factorial design indicating significant factors influencing particle size. (B) Pareto chart from the CCD. (C) Response surface model illustrating the interaction between temperature and NH_4_OH flow rate.

The CCD results showed that the NH_4_OH flow rate was the dominant factor in controlling the particle size, with the optimal value identified at 5.5 mL·min^–1^. Although temperature exhibited a comparatively smaller effect, it still influenced particle size, with the ideal condition observed at approximately 65 °C. These findings align with previous reports. For instance, Pei et al. (2007) demonstrated that elevated temperatures favor the formation of monodisperse, superparamagnetic nanoparticles with average diameters around 11 nm, attributed to enhanced crystal growth dynamics under thermal activation.^46^ Similarly, Mascolo et al. (2013) highlighted that rapid basic solution addition promotes continuous nucleation over particle growth, thereby producing smaller nanoparticles.^47^ While the optimal flow rate identified in this study differs slightly from the 1.88 mL·min^–1^ reported by Ahn et al. (2012), this variation is likely due to differences in experimental setup and reagent concentrations.^48^

Overall, the design of experiments (DoE) approach proved to be effective in optimizing synthesis conditions. By narrowing down the most influential parameters and refining their levels, the study established a reproducible route for obtaining nanoparticles with reduced size and a more uniform distribution. These aspects are key attributes for enhancing surface interactions in downstream applications, such as nucleic acid extraction.

### 3.2 Characterization of Nanoparticles

Nanoparticles synthesized at the optimal point of the CCD (temperature: 65 °C; NH_4_OH flow rate: 5.5 mL·min^–1^) were characterized to confirm their structure, morphology, size distribution, and surface properties.

XRD analysis (Figure 2A) confirmed the crystalline nature of the Fe_3_O_4_ nanoparticles. Distinct diffraction peaks were observed at 2θ values of 30.1°, 35.5°, 42.6°, and 62.8°, corresponding to the (220), (311), (400), and (440) planes, respectively. These values are consistent with the inverse spinel cubic structure of magnetite.^40,46,49,50^ This crystalline pattern confirms that the desired phase was successfully achieved under the selected synthesis conditions.

**Figure 2:**
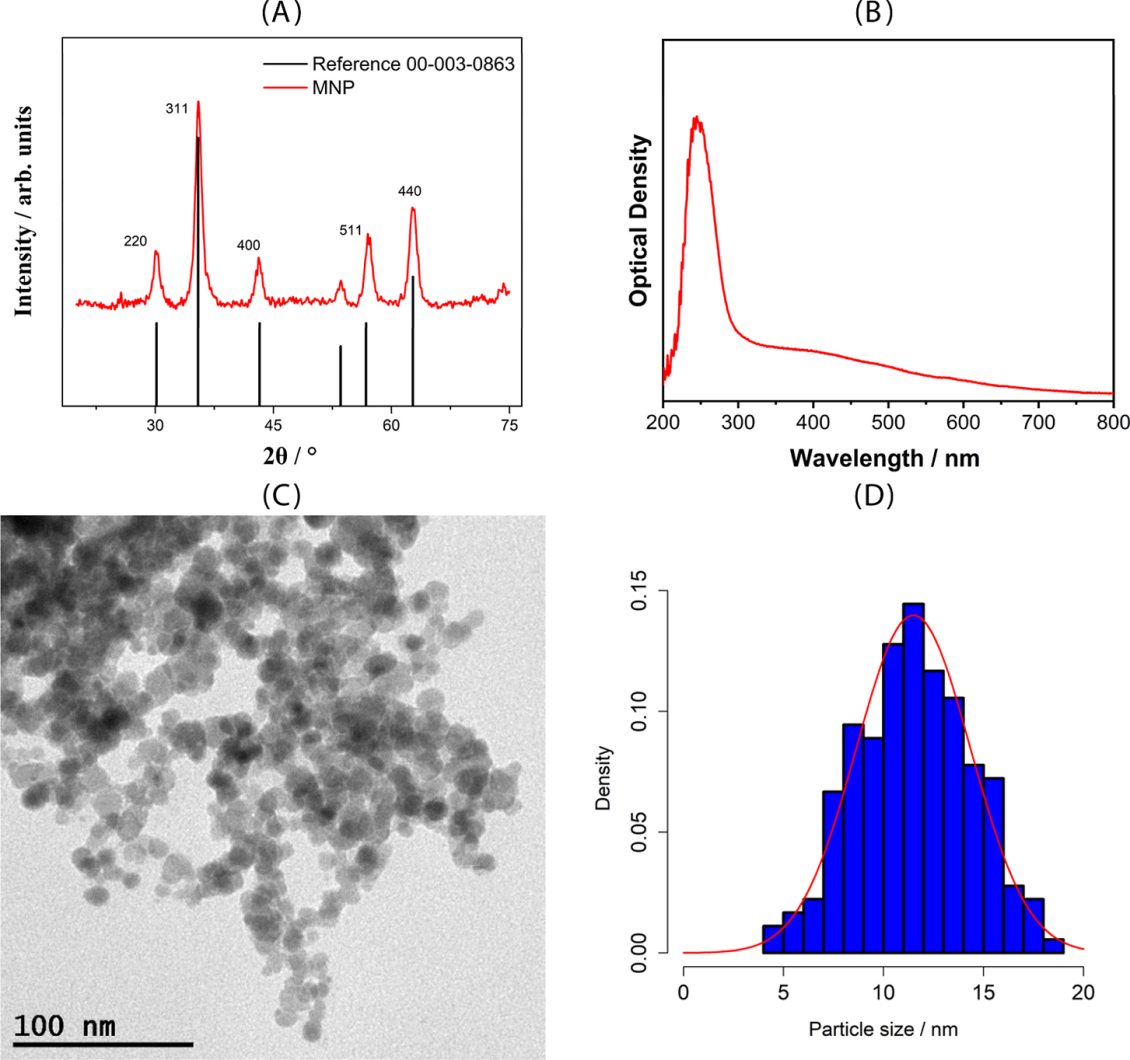
Physicochemical characterization of the MNPs: (A) XRD pattern (B) UV/vis; (C) TEM micrography of MNPs; (D) Size distribution histogram obtained by measuring the diameters of approximately 200 individual particles from TEM images.

The UV–Vis absorption spectrum (Figure 2B) shows a broad band between 330 and 450 nm and a strong band around 260 nm. The band at 260 nm is typically related to electronic transitions in magnetite, and the broad band in the visible range is also commonly observed for Fe_3_O_4_ due to charge transfer between iron ions.^51^ This feature is commonly attributed to plasmonic resonance and interband electronic transitions in Fe_3_O_4_, supporting the formation of magnetic nanoparticles.^52,53^

TEM images (Figure 2C) revealed that the MNPs were predominantly spherical and well-dispersed with an average diameter of 11.49 ± 2.85 nm (Figure 2D). This value was obtained by measuring the diameters of approximately 200 individual particles within a range of 4 to 18 nm, indicating a relatively narrow size distribution. This feature is highly desirable, particularly in biomedical and analytical applications, where consistency in particle size enhances performance and reproducibility. These findings are consistent with previous studies; for example, Radoń et al. (2017) also reported the synthesis of superparamagnetic nanoparticles with an average size of 12 nm using a similar coprecipitation method.^54^

ZP of naked MNP indicated a surface charge of −31 mV. This high negative charge is likely due to the presence of abundant surface hydroxyl groups (−OH) on the magnetite surface. Typically, nanoparticles with ZP values greater than +25 mV or less than −25 mV are considered stable in suspension. Thus, the synthesized MNPs demonstrate exceptional stability, which is advantageous for their use in environmental and biomedical applications.^55,56^ After modification with TEOS and APTES, the ZP increases to −16 mV due to the presence of positively charged −NH_2_ groups which is crucial for DNA extraction. Figure S1 shows the ZP measured at each step of synthesis and functionalization of MNPs.

The results from XRD, UV/vis, TEM, DLS, and ZP analyses confirm the successful synthesis of MNP with desirable structural integrity, monodispersity, and colloidal stability. These properties are critical for ensuring reliable performance in subsequent functionalization steps and practical applications, such as nucleic acid extraction.

To confirm the modification of MNPs with TEOS and APTES, the vibrational profile of MNPs was analyzed through IR and Raman spectroscopy. Figure 3A presents the IR spectrum of the Fe_3_O_4_@Si@APTES nanoparticles. The bands centered at 875 cm^–1^ and 1040 cm^–1^, can be assigned to chemical bond vibrations of Si–O–H bending and Si–O–Si stretching, respectively.^57,58^ Figure 3B presents the Raman spectrum of Fe_3_O_4_@Si@APTES, the 480 cm^–1^ signal can be related to Fe_3_O_4_, and bands at 1330 cm^–1^, 1480 cm^–1^, and 1660 cm^–1^ could be related to the APTES ligand.^59–61^ Thus, after highly stable magnetite nanoparticles with proven functionalization by TEOS and APTES ligands were obtained, the nanoparticles were applied to extract and purify nucleic acids.

**Figure 3:**
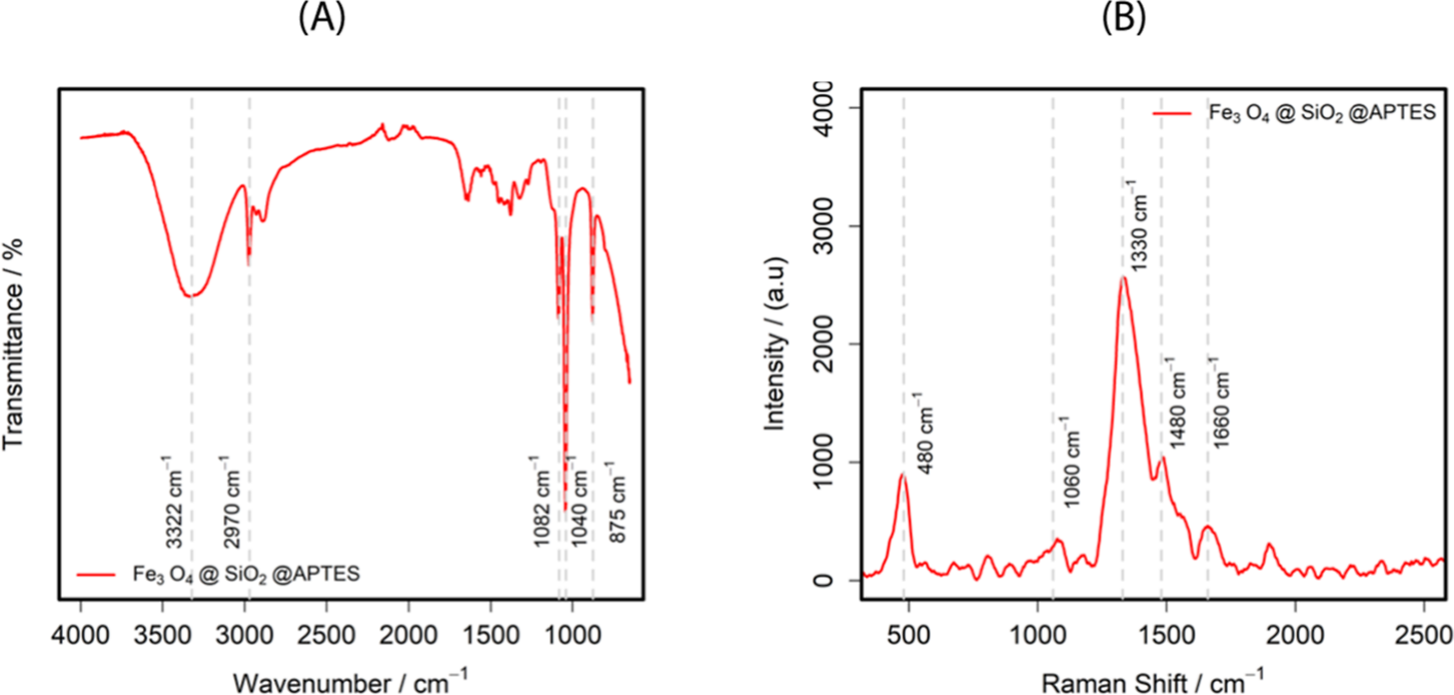
(A) IR spectrum and (B) Raman spectrum of Fe_3_O_4_@Si@APTES.

### 3.3 pH Responsive Surface Charge of Fe_3_O_4_@Si@APTES MNPs

The pH-dependent ZP measured at 20 °C is shown in Figure 4A. The surface charge of MNPs remains negative at pH 8 to 10 due to deprotonated functional groups. The negative charge decreased with decreasing from pH 8 to 4, becoming very positive at pH 4, which is ascribable to protonation of −NH_2_ groups and free −OH present on the surface of nanoparticles in aqueous suspension.

**Figure 4:**
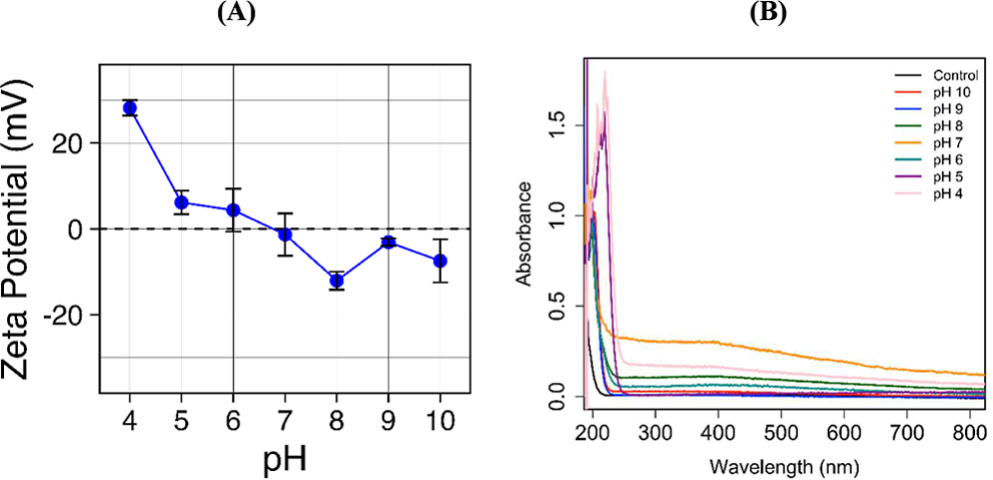
(A) pH-dependent ZP of Fe_3_O_4_@Si@APTES MNPs and (B) UV/vis spectra of aqueous solution of Fe_3_O_4_@Si@APTES MNPs after magnetization at different pH conditions.

The colloidal stability of Fe_3_O_4_@Si@APTES nanoparticles under different pH conditions was monitored through UV/vis spectroscopy. Upon destabilization in solution, nanoparticles tend to aggregate, reducing mobility under an applied magnetic field and increasing the sedimentation.^62^ Figure 4B shows the UV/vis spectra of the aqueous solution after 5 s of magnetization of the nanoparticles. No significant changes were observed in absorption spectra at the pH range of 8 to 10, indicating relatively stable nanoparticles. However, at lower pH levels, the background of UV/vis spectra enhances, which could be ascribed to the formation of nanoparticle aggregates due to the change from negative to positive surface charge.

The ZP dependence of temperature and ionic strength was also evaluated. It seems that the increasing temperature does not significantly affect the nanoparticle surface charge (Figure 5A). However, the UV/vis data of the aqueous solution after 5 s of magnetization shows a slight change of spectral profile of Fe_3_O_4_@Si-APTES at 60 °C, which indicates fewer magnetized particles and loss of stability or aggregation (Figure 5C). Figure 5B shows the effect of the ionic strength on the surface charge of magnetic nanoparticles; two different regimes were observed, and at low salt concentrations (10 mM to 50 mM), the increase of ionic strength decreases the absolute value of ZP due to the screening of the electric double layer and weakens the electrostatic repulsion.^63^ From 100 to 200 mM, the magnetic nanoparticles become more negative. However, high salt concentration strongly increases the conductivity of the medium and makes the ZP measurements less reliable. Despite the different behaviors observed in ZP,^64^ UV/vis (Figure 5D) indicates no significant effect of ionic strength over nanoparticle magnetic stability.

**Figure 5:**
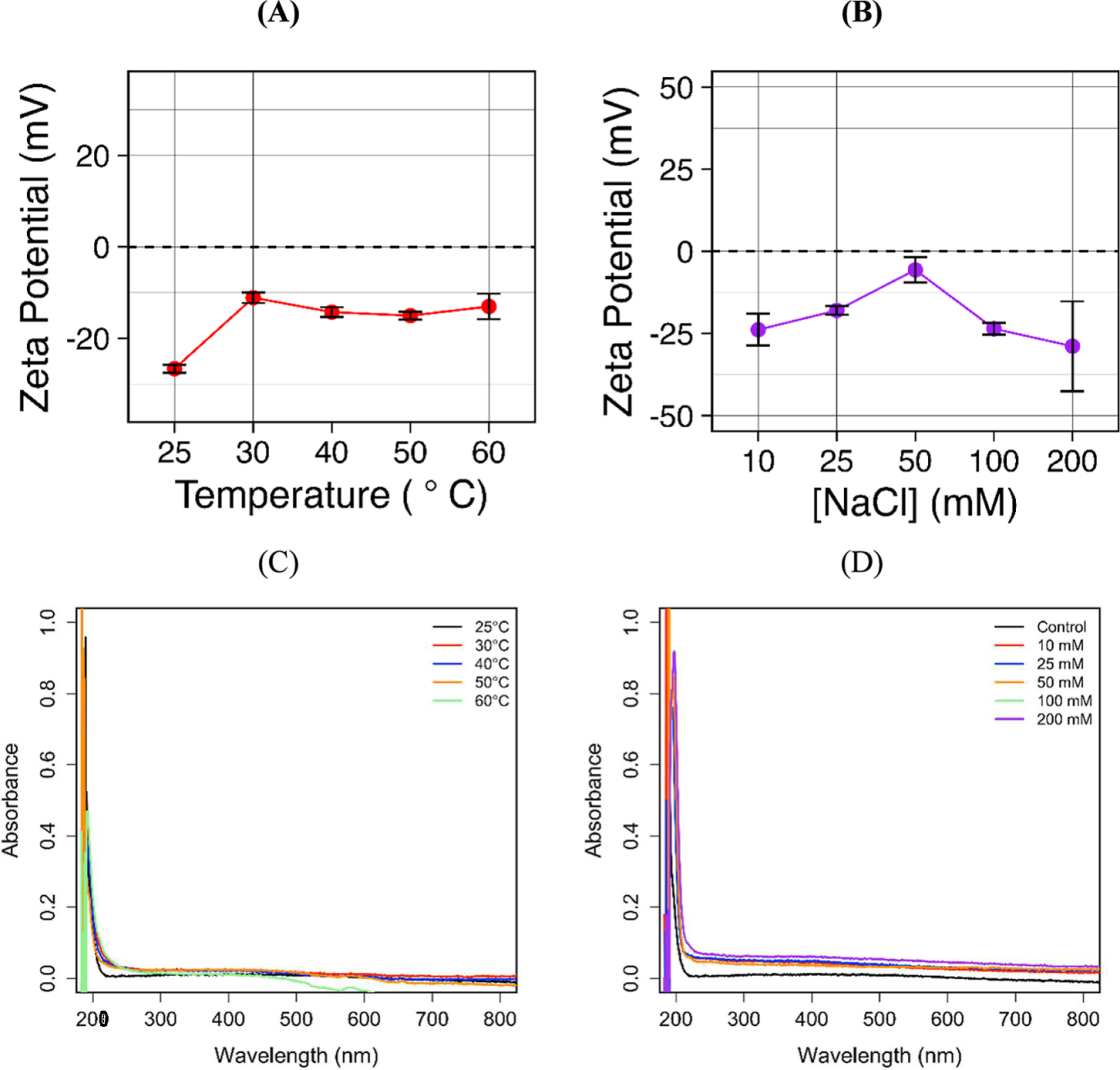
(A) Temperature-dependent and (B) ionic strength ZP of Fe_3_O_4_@Si-APTES magnetic nanoparticles. UV/vis spectra of aqueous solution of Fe_3_O_4_@Si-APTES MNPs after magnetization at (A) different temperatures and (D) ionic strengths.

### 3.4 Extraction and Purification of Nucleic Acids

In this study, TEOS was primarily employed to generate a uniform silica shell around the iron oxide nanoparticles, acting as a protective and stabilizing layer that improves colloidal stability and prevents aggregation, as previously demonstrated in similar silica-coating strategies.^65,66^ Although the resulting silica surface exposes silanol (−OH) groups, these functionalities provide only limited affinity toward nucleic acids, since they lack strong specific interactions with the phosphate backbone.^67^ To enhance the binding capacity, the nanoparticles were further modified with APTES, which covalently anchors to the silica layer and introduces terminal amino (−NH_2_) groups, and the −NH_2_ density obtained from eq 1 was 1.25 mmol of −NH_2_ per 1 g of MNP.^41^ These amine groups establish favorable electrostatic interactions with the negatively charged phosphate backbone of nucleic acids, thereby improving binding affinity and extraction efficiency.^17,68^ The superior performance of APTES-functionalized nanoparticles compared to TEOS-only coated particles can thus be attributed to the presence of surface amines, which create a more effective chemical interface for nucleic acid adsorption and recovery, in line with reports showing that amino-modified magnetic nanoparticles outperform bare silica coatings in nucleic acid isolation workflows.^68,69^

The applicability of Fe_3_O_4_@SiO_2_-APTES nanoparticles for the extraction and purification of NAs was evaluated by comparing their performance to two widely used methods: (i) manual extraction using a silica column (Bio-Gene DNA/RNA Extraction Kit, Bioclin) and (ii) automated extraction with commercial magnetic beads (Extracta Kit Fast DNA and RNA VIRAL, Loccus Kit). To ensure comparability, the experimental protocol for Fe_3_O_4_@SiO_2_-APTES closely followed the buffer compositions and extraction steps used in the automated kit.

Quantitative assessment of extraction efficiency was performed using RT-qPCR, a gold-standard technique for detecting viral RNA. The cycle threshold (Ct) value was used as the main readout. This value represents the number of amplification cycles required for the fluorescent signal to exceed a predefined threshold above the background level. Because the fluorescence increases with the accumulation of the amplified product, samples with higher initial RNA concentrations reach the threshold in fewer cycles, resulting in lower Ct values. Therefore, a lower Ct indicates more efficient RNA extraction and purification.^70,71^

To confirm the presence of SARS-CoV-2 RNA in clinical saliva samples, RT-qPCR targeted three specific viral genes: N, S, and Orf1ab. These genes were selected because they represent conserved regions of the viral genome and are commonly used in diagnostic kits for SARS-CoV-2 detection. Amplification of multiple genes increases the specificity and reliability of the assay.^72^

As shown in Figure 6, the amplification curves illustrate the quantification cycles obtained for each gene from RNA extracted by using Fe_3_O_4_@SiO_2_-APTES nanoparticles. The Ct values observed ranged within the expected diagnostic window (typically 17–37 cycles). The relatively low Ct values confirmed the nanoparticles’ capability to efficiently extract and purify RNA, enabling successful downstream amplification. This performance highlights the potential of Fe_3_O_4_@SiO_2_-APTES as an effective alternative to conventional extraction platforms, especially in contexts where cost, scalability, and automation are limiting factors.

**Figure 6:**
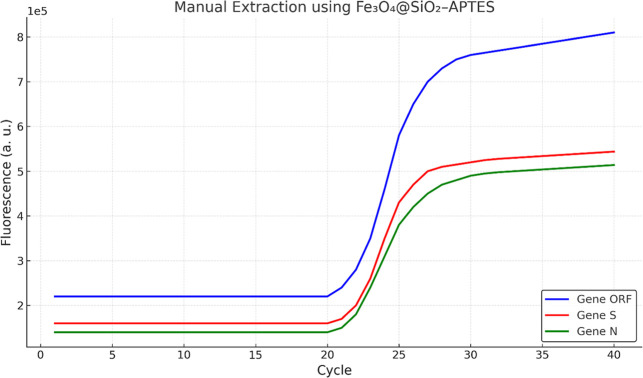
Curves corresponding to the quantification cycles of nucleic acids obtained through manual extraction using the nanoparticles of Fe_3_O_4_@SiO_2_-APTES.

A comparison of the Ct values for nucleic acids (NA) extracted using the silica column method and Fe_3_O_4_@SiO_2_-APTES nanoparticles (Figure 7A) revealed no statistically significant differences, indicating a comparable extraction efficiency between the two approaches. Similarly, the performance of the Fe_3_O_4_@SiO_2_-APTES nanoparticles did not differ significantly from that of the commercial automated kit employing magnetic beads (Figure 7B). These findings demonstrate the potential of the synthesized nanoparticles as viable alternatives for NA extraction and purification. However, it is important to acknowledge that the experimental protocol was deliberately aligned with the conditions of the commercial kit, including reagent composition and processing steps. While this facilitates a controlled comparison, it may not reflect the full optimization potential of the nanoparticle system. Future studies should focus on tailoring buffer composition and process parameters specifically for the Fe_3_O_4_@SiO_2_-APTES platform to enhance yield, selectivity, and purity. Additionally, broader validation using diverse clinical matrices and target genes is essential to confirm the robustness and applicability of the proposed approach in diagnostic settings.

**Figure 7:**
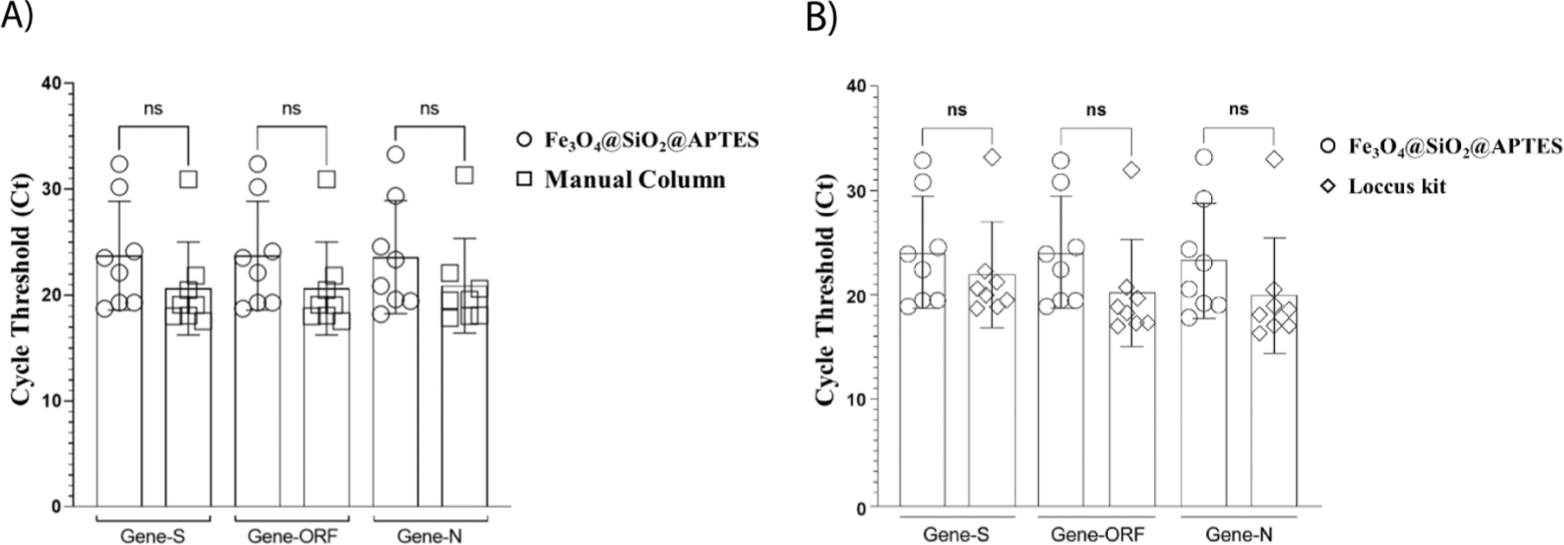
Comparison of Ct values obtained during RT-qPCR analysis using different RNA extraction methods. (A) Manual extraction using a silica-based spin column versus extraction with Fe_3_O_4_@SiO_2_-APTES nanoparticles. (B) Automated extraction using commercial magnetic beads (Loccus kit) versus extraction with Fe_3_O_4_@SiO_2_-APTES nanoparticles. Differences were assessed by statistical analysis; “ns” indicates nonsignificant differences (*p* > 0.05). Statistical analysis was performed by comparing the extraction methods (Fe_3_O_4_@SiO_2_@APTES vs Manual Column and Fe_3_O_4_@SiO_2_@APTES vs Automated extraction–Loccus) for each gene (S, ORF, N) using independent-samples *t* tests. No significant differences were observed (all *p* > 0.05). The corresponding table with these results has been included in the Supporting Information (Table S8).

Ct values obtained through RT-qPCR serve as a functional metric that simultaneously reflects yield, purity, and amplification compatibility.^70^ Comparable to other studies on PEI-, GO-, and imidazolium-modified MNPs that also emphasize Ct-based performance assessment,^17^ our findings confirm that Fe_3_O_4_@SiO_2_-APTES nanoparticles perform at a similar level to state-of-the-art extraction platforms.

Magnetic iron oxide nanoparticles have long been explored for nucleic acid (NA) extraction due to their unique magnetic responsiveness and surface tunability. Chacón-Torres et al. (2020) reported a scalable and simplified synthesis of MNPs coated with APTES for RNA extraction, confirming functional amine groups but lacking detailed size and stability optimization that our study achieves via DoE.^73^ Additionally, Ali et al. (2022) developed sugar-based cationic core–shell silica MNPs functionalized with imidazolium groups demonstrating excellent nucleic acid loading efficiencies, yet with less emphasis on synthesis parameter optimization and performance validation in inhibitor-rich, low-input samples.^24^ Ma et al. (2013) demonstrated the use of TEOS-functionalized nanoparticles (∼500 nm) for the successful isolation of DNA from diverse biological matrices, including bacterial, yeast, blood, and viral samples, achieving high yields suitable for downstream applications.^74^ Bai et al. (2016) further emphasized the importance of surface chemistry, showing that amine-rich nanoparticles significantly enhanced DNA binding efficiency.^75^ These findings align with the results of the present study, wherein Fe_3_O_4_@SiO_2_-APTES nanoparticles—bearing a high density of amine groups—exhibited extraction efficiency comparable to that of commercial kits. The favorable performance observed in this study highlights the critical role of surface functionalization in enabling efficient nucleic acid extraction and demonstrates the feasibility of implementing such nanomaterials in clinical and decentralized molecular diagnostic settings. Importantly, the material also exhibits a very low cost per extraction, approximately USD 0.017, as shown in Table S7, which further strengthens its practical applicability. Taken together, these results validate the design of Fe_3_O_4_@SiO_2_-APTES nanoparticles as a cost-effective, scalable, and high-performance platform for nucleic acid purification with significant potential for future integration into point-of-care and high-throughput diagnostic workflows.

## 4 Conclusions

This study demonstrated that the synthesis of magnetic iron oxide nanoparticles via coprecipitation can be precisely controlled by adjusting the NH_4_OH flow rate and reaction temperature, with optimal conditions identified at 5.5 mL·min^–1^ and 65 °C. These parameters led to the formation of monodisperse Fe_3_O_4_ nanoparticles with an average diameter of 12 nm. Subsequent functionalization with TEOS and APTES successfully conferred colloidal stability and introduced silane and amine surface groups, which are key features for biomolecular interactions. When applied to NA extraction, the Fe_3_O_4_@SiO_2_–APTES nanoparticles achieved performance comparable to that of established commercial kits, as evidenced by Ct values between 20 and 25 for clinically relevant genes (S, ORF, and N). These findings highlight the potential of this nanomaterial as a cost-effective and scalable alternative to NA purification workflows. Overall, this platform offers a promising foundation for the development of next-generation magnetic supports for molecular diagnostics, particularly in resource-limited and point-of-care settings. In addition to demonstrating diagnostic performance comparable to existing solutions, it distinguishes itself by relying on inexpensive reagents (FeCl_2_, FeCl_3_, NH_4_OH, TEOS, and APTES) and straightforward, scalable synthetic steps—features that reinforce its cost-effectiveness when compared to commercial kits.

## Supplementary Material



## Data Availability

The data supporting this article have been included in the main text. Additional raw data required to reproduce these findings can be made available upon reasonable request to the author.

## Notes

ZP measurements at different stages of magnetic nanoparticle functionalization; bibliographic review of synthesis parameters influencing magnetic nanoparticle size; detailed protocols for viral RNA extraction using manual silica columns and automated magnetic bead-based systems; complete results from fractional factorial and central composite experimental designs; ANOVA analyses, response surface data, and hydrodynamic diameter measurements; cost analysis of reagents; and statistical comparisons between nucleic acid extraction methods to assess performance and reproducibility (PDF)
