# Supplementary material for: From Synthesis to Application: Functionalized Magnetic Nanoparticles as a Simple and Reliable Tool for Nucleic Acid Purification
Source: ACS Omega. 2026 Jan 21;11(4):5063–74. doi: 10.1021/acsomega.5c06432 (PMC12878784; doi:10.1021/acsomega.5c06432)
Supplement: Supplementary file 1 [file ao5c06432_si_001.pdf]

Supplementary material

From Synthesis to Application: Functionalized Magnetic Nanoparticles as a Simple and Reliable Tool for Nucleic Acid Purification

Iuly Guimarães Ribeiro[1#], Thais de Andrade Silva[1,2#], Ana Carolina de Lima Barizão[1,2], Giordano Toscano Paganoto[1], Gabriel Fernandes Souza dos Santos[1], Sérvio Tulio Alves Cassini[2], Marco Cesar Cunegundes Guimarães[1], Jairo Pinto de Oliveira[1,2*]

[1] Federal University of Espírito Santo, Campus Maruípe, Av. Marechal Campos 1468, Vitória- ES, 29.040-090, Brazil
[2] Center of Research, Innovation and Development, Laboratory of Environmental Characterization, Cariacica - ES, 29.140-130, Brazil.
[#] Both authors contributed equal in this article as first author
*Corresponding author: jairo.oliveira@ufes.br

Figure S1. Zeta potential measurement of each step of MNP modification

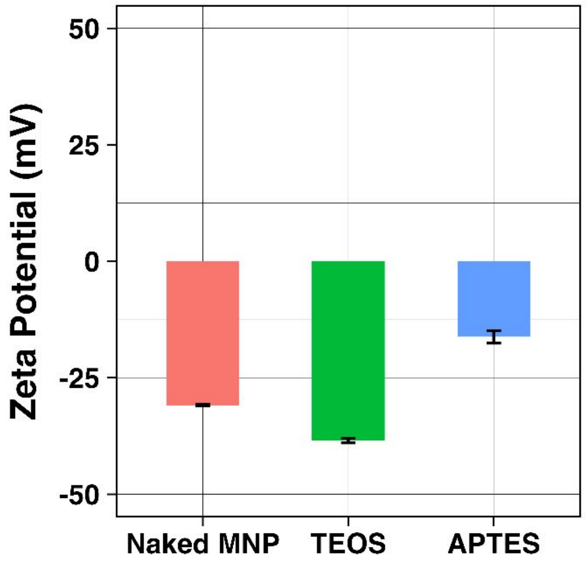

Table S1. Bibliographic review of the main variables and their levels interfering with the size of magnetic nanoparticles.

**Metalic Salt**	**Experimental Conditions**	**Nanoparticle size**	**Ref.**
$FeCl_3$, $FeCl_2$	2:1 / 1 h / 0.7 ml.min^{-1} / 90°C	16–40 nm	[1]
$FeCl_3$, $FeCl_2$	0.8:1 / 170°C / 1h	10 nm	[2]
$FeCl_3$, $FeCl_2$	2:1/ 2.000 rpm / pH 10 / 10 min. / 26.85°C	11 nm	[3]
$FeCl_3$, $FeCl_2$	2:1 / 450 rpm / nitrogen / 25 min. / 25ºC	16 nm	[4]
$FeCl_3$, $FeCl_2$	1:1 / 500 rpm / 25ºC	30 nm	[5]
$FeCl_3$, $FeCl_2$	2:1 / 80°C / 1h / nitrogen.	7.3 nm	[6]
$FeCl_3$, $FeCl_2$	2:1 / 1.200 rpm / 25°C / 30 min	10 nm	[7]
$FeCl_3$, $FeCl_2$	2:1 / 300 rpm / 30 min. / 75°C	11 nm	[8]
$FeCl_3$, $FeCl_2$	2:1 / 60°C / 30 min / vigorous stirring	8.3 nm	[9]
$FeCl_3$, $FeCl_2$	2:1 / 1h / 70°C / 700 rpm / 0.2 ml min^{-1}	13 nm	[10]
$FeCl_3$, $FeCl_2$	2:1 / 1.100 rpm	7.5 nm	[11]
$FeCl_3$, $FeCl_2$	2:1 / 1h / 90°C / argon	50 nm	[12]
$FeCl_3$, $FeCl_2$	2:1 / 25°C / hard agitation	25 nm	[13]
$FeCl_3$, $FeCl_2$	2:1 / 80ºC / 30 min.	10.4 nm	[14]
$FeCl_3$, $FeCl_2$	2:1 / 80°C / 800 rpm / 30 min.	12 nm	[15]
$FeCl_3$, $FeCl_2$	2:1 / 90°C / 30 min.	20 nm	[16]
$FeCl_3$, $FeCl_2$	2:1 / 80°C / 20 min. / hard agitation	40 nm	[17]
$FeCl_3$, $FeCl_2$	2:1 / 60°C/ 10 min.	20 – 40 nm	[18]

Table S2. Detailed protocols for viral RNA extraction from saliva. The table summarizes the reagents, volumes, and steps used for manual silica column extraction and automated magnetic bead-based extraction (Loccus system), including lysis, binding, washing, elution, and Proteinase K treatment. The extracted RNA was used as template in PCR reactions.

Method	Protocol
Extraction of Nucleic Acids Using Silica Column	For viral RNA extraction from saliva using the silica column kit, 200 µL of sample were added to a 1.5 mL tube at room temperature, followed by 5 µL of Proteinase K and homogenization. Then, 200 µL of lysis buffer and 5.6 µL of Carrier RNA were added, mixed vigorously, and incubated for 3 minutes at room temperature. Next, 200 µL of ethanol (96–100%) were added, vortexed, and incubated for 5 minutes at room temperature.<br><br>The total volume (~610 µL) was transferred to the silica column and centrifuged for 3 minutes at 4,000 × g. If the column was not completely dry, a second centrifugation at ~15,000 × g was performed. The column was then transferred to a new collection tube.<br><br>RNA bound to the column was washed in three steps: 400 µL of the first wash reagent centrifuged for 1 minute at 11,000 × g, followed by 400 µL of the second wash reagent centrifuged for 1 minute at 11,000 × g, and finally 200 µL of the second wash reagent centrifuged for 5 minutes at ≥15,000 × g.<br><br>For elution, the column was transferred to a clean tube and incubated for 5 minutes at 56°C with the lid open. Then, 30–60 µL of RNase-free water preheated to 70°C were added to the membrane, incubated at room temperature for 3 minutes, and centrifuged for 3 minutes at ≥15,000 × g. The column was discarded, and the eluted RNA was used as template in PCR.
Extraction of Nucleic Acids Using Automated Extraction (Loccus)	For automated viral RNA extraction from saliva using the Loccus system with magnetic beads, 10 µL of Proteinase K (20 mg mL^{-1}) were added to each well of columns 1 and 7 (rows A–H), followed by 200 µL of saliva per well (one well per sample).<br><br>The system performed lysis, binding, washing, and elution automatically. Reagent volumes per column were: 600 µL lysis buffer, 800 µL Wash Buffer 1, 800 µL Wash Buffer 2 (twice), 800 µL magnetic beads, and 80 µL elution buffer. Magnetic beads captured the RNA during the automated washes, and RNA was eluted in 80 µL elution buffer. The extracted RNA was then used as template in PCR.

Table S3. Fractional factorial results $2^{(5-1)}$.

	Factor					Response variable	
**Treatment**	**Time (min.)**	**Temperature (°C)**	**Molar Ratio ($FeCl_2$:$FeCl_3$)**	**Stirring (RPM)**	**NH_4OH flow ($ml.min^{-1}$)**	**Diameter (nm)**	**Hydrodynamic diameter (nm)**
**1**	10	25	1	200	10	7.039	35.901
**2**	180	25	1	200	1	8.315	33.153
**3**	10	100	1	200	1	9.359	30.302
**4**	180	100	1	200	10	10.717	27.625
**5**	10	25	3	200	1	7.244	26.568
**6**	180	25	3	200	10	8.330	41.363
**7**	10	100	3	200	10	8.973	22.909
**8**	180	100	3	200	1	9.424	27.954
**9**	10	25	1	800	1	6.716	33.872
**10**	180	25	1	800	10	9.933	40.361
**11**	10	100	1	800	10	12.362	25.517
**12**	180	100	1	800	1	13.072	29.865
**13**	10	25	3	800	10	8.153	41.263
**14**	180	25	3	800	1	7.232	25.896
**15**	10	100	3	800	1	6.667	20.669
**16**	180	100	3	800	10	5.821	25.187

Table S4. Response obtained from the face-centered CCD.

Treatment	Temperature (°C)	NH_4OH flow (ml min^{-1})	Hydrodynamic diameter (nm)
1	30 (−)	0.5 (−)	158.39
2	30 (−)	5.5 (0)	165.32
3	30 (−)	10 (+)	262.57
4	65 (0)	0.5 (−)	137.87
5	65 (0)	5.5 (0)	88.21
6	65 (0)	10 (+)	236.62
7	100 (+)	0.5 (−)	99.56
8	100 (+)	5.5 (0)	73.44
9	100 (+)	10 (+)	181.00
10	65 (0)	5.5 (0)	128.32
11	65 (0)	5.5 (0)	156.63

Table S5. Analysis of Variance (ANOVA) from the fractional factorial $2^{(5-1)}$. Bold values indicate significant variables.

Variable	SS	df	MSQ	F	p
Time (min)	12.9639145	1	12.9639145	0.562426655	0.470560159
**Temperature (ºC)**	**291.974612**	**1**	**291.974612**	**12.6670308**	**0.00518921393**
Molar ratio	38.3966159	1	38.3966159	1.66579934	0.22586336
Stirring(RPM)	0.618530495	1	0.618530495	0.026834336	0.873142733
NH_4OH flow (mL min^{-1})	63.3831488	1	63.3831488	2.74981544	0.128257375
Error	230.499647	10	23.0499647		
Total SS	637.836469	15			

Table S6. Analysis of Variance (ANOVA) from the face-centered CCD considering temperature and NH_4OH flow variables. Bold values indicate significant variables 3^2.

Variables	SS	df	MSQ	F	p
**Temperature (ºC) (L)**	**8991.67**	**1**	**8991.67**	**20.04686**	**0.004204**
Temperature (ºC) (Q)	188.00	1	188.00	0.41915	0.541325
**NH_4OH flow (mL min^{-1}) (L)**	**13478.02**	**1**	**13478.02**	**30.04916**	**0.001541**
**NH_4OH flow (mL min^{-1}) (Q)**	**8892.50**	**1**	**8892.50**	**19.82578**	**0.004317**
Error	2691.19	6	448.53		
Total SS	34194.16	10			

Table S7. Cost analysis performed in October 2025 based on high-purity Sigma-Aldrich standard reagents. Values expressed in US dollars.

Material	Quantity (unit)	Price (USD)	Amount Used (USD)	# Extractions	Cost per Extraction (USD)
$FeCl_3$ (Sigma Aldrich F2877)	0.03 mol·L^{-1}; 10 mL	500 mL / USD 56.70	1.134	600	0.0019
$FeCl_2$ (Sigma Aldrich 44939)	0.06 mol·L^{-1}; 10 mL	1000g / USD 316.00	3.16	600	0.0053
NH_4OH 28% (Sigma Aldrich 221228)	5.3 mL	1000mL / USD 77.50	0.41	600	0.00068
TEOS (Sigma Aldrich 86578)	0.35 mL	1000mL / USD 146.00	0.05	600	0.000083
APTS (Sigma Aldrich 440140)	2.26 mL	500 mL / USD 380.00	0.71	600	0.0012
Ethanol (Sigma Aldrih E7023)	30 mL	1000mL / USD 157.00	4.75	600	0.0079
N_2 (White Martins 99,99%)	0,001 m3	10 m^3 / USD 120	0.12	600	0.00020
				**Total**	0.017

Table S8. Statistical analysis comparing the extraction methods (Fe_3O_4@SiO_2@APTES vs. Manual Column and Fe_3O_4@SiO_2@APTES vs. Automated extraction – Loccus) for each gene (S, ORF, N) using independent-samples t-tests. No significant differences were observed (all $p > 0.05$).

**Fe_3O_4@SiO_2@APTES x Manual Column**								
	**Significant?**	**P value**	**Mean1**	**Mean2**	**Difference**	**SE of difference**	**t ratio**	**df**
Gene S	ns	0,722891	23,9	24,75	-0,85	2,30225	0,369204	7,0
Gene ORF	ns	0,681057	22,6667	23,5	-0,833333	1,95434	0,426401	8,0
Gene N	ns	0,782713	23,0	23,5	-0,5	1,75297	0,28523	8,0
**Fe_3O_4@SiO_2@APTES x Automated extraction (Loccus)**								
	**Significant?**	**P value**	**Mean1**	**Mean2**	**Difference**	**SE of difference**	**t ratio**	
Gene S	ns	0,740979	27,0778	26,94	0,137778	0,410047	0,336005	17,0
Gene ORF	ns	0,483749	27,93	27,63	0,3	0,41955	0,715051	18,0
Gene N	ns	0,346486	26,64	26,24	0,4	0,413763	0,966737	18,0

References

[1] Wu S, Sun A, Zhai F, Wang J, Xu W, Zhang Q, *et al.* Fe3O4 magnetic nanoparticles synthesis from tailings by ultrasonic chemical co-precipitation. Mater Lett 2011;65:1882–4. https://doi.org/10.1016/J.MATLET.2011.03.065.

[2] Wu W, Wu Z, Yu T, Jiang C, Kim WS. Recent progress on magnetic iron oxide nanoparticles: Synthesis, surface functional strategies and biomedical applications. Sci Technol Adv Mater 2015;16:023501. https://doi.org/10.1088/1468-6996/16/2/023501.

[3] F. Hasany S, Ahmed I, J R, Rehman A. Systematic Review of the Preparation Techniques of Iron Oxide Magnetic Nanoparticles. Nanoscience and Nanotechnology 2013;2:148–58. https://doi.org/10.5923/j.nn.20120206.01.

[4] Laurent S, Dutz S, Häfeli UO, Mahmoudi M. Magnetic fluid hyperthermia: Focus on superparamagnetic iron oxide nanoparticles. Adv Colloid Interface Sci 2011;166:8–23. https://doi.org/10.1016/J.CIS.2011.04.003.

[5] Akutsu JI, Tojo Y, Segawa O, Obata K, Okochi M, Tajima H, *et al.* Development of an integrated automation system with a magnetic bead-mediated nucleic acid purification device for genetic analysis and gene manipulation. Biotechnol Bioeng 2004;86:667–71. https://doi.org/10.1002/BIT.20049.

[6] Rajan S, Venugopal A, Kozhikkalathil H, Valappil S, Kale M, Mann M, *et al.* Synthesis of ZnO nanoparticles by precipitation method: Characterizations and applications in decipherment of latent fingerprints. Mater Today Proc 2023. https://doi.org/10.1016/J.MATPR.2023.05.680.

[7] Kaushik P, Malik A. Process optimization for efficient dye removal by Aspergillus lentulus FJ172995. J Hazard Mater 2011;185:837–43. https://doi.org/10.1016/J.JHAZMAT.2010.09.096.

[8] Tuzen M, Sarı A, Saleh TA. Response surface optimization, kinetic and thermodynamic studies for effective removal of rhodamine B by magnetic AC/CeO2 nanocomposite. J Environ Manage 2018;206:170–7. https://doi.org/10.1016/J.JENVMAN.2017.10.016.

[9] Van Thuan T, Quynh BTP, Nguyen TD, Ho VTT, Bach LG. Response surface methodology approach for optimization of Cu2+, Ni2+ and Pb2+ adsorption using KOH-activated carbon from banana peel. Surfaces and Interfaces 2017;6:209–17. https://doi.org/10.1016/J.SURFIN.2016.10.007.

[10] Thangaraj B, Jia Z, Dai L, Liu D, Du W. Effect of silica coating on Fe3O4 magnetic nanoparticles for lipase immobilization and their application for biodiesel production. Arabian Journal of Chemistry 2019;12:4694–706. https://doi.org/10.1016/J.ARABJC.2016.09.004.

[11] Pei W, Kumada H, Natusme T, Saito H, Ishio S. Study on magnetite nanoparticles synthesized by chemical method. J Magn Magn Mater 2007;310:2375–7. https://doi.org/10.1016/J.JMMM.2006.10.837.

[12] Mascolo MC, Pei Y, Ring TA. Room Temperature Co-Precipitation Synthesis of Magnetite Nanoparticles in a Large pH Window with Different Bases. Materials

2013, Vol 6, Pages 5549-5567 2013;6:5549–67. https://doi.org/10.3390/MA6125549.

[13] Ahn T, Kim JH, Yang HM, Lee JW, Kim JD. Formation pathways of magnetite nanoparticles by coprecipitation method. Journal of Physical Chemistry C 2012;116:6069–76. https://doi.org/10.1021/JP211843G/SUPPL_FILE/JP211843G_SI_001.PDF.

[14] Rahman OU, Mohapatra SC, Ahmad S. Fe3O4 inverse spinal super paramagnetic nanoparticles. Mater Chem Phys 2012;132:196–202. https://doi.org/10.1016/J.MATCHEMPHYS.2011.11.032.

[15] Radoń A, Drygała A, Hawełek Ł, Łukowiec D. Structure and optical properties of Fe3O4 nanoparticles synthesized by co-precipitation method with different organic modifiers. Mater Charact 2017;131:148–56. https://doi.org/10.1016/J.MATCHAR.2017.06.034.

[16] Shukla S, Jadaun A, Arora V, Sinha RK, Biyani N, Jain VK. In vitro toxicity assessment of chitosan oligosaccharide coated iron oxide nanoparticles. Toxicol Rep 2015;2:27–39. https://doi.org/10.1016/J.TOXREP.2014.11.002.

[17] Chang YC, Chen DH. Preparation and adsorption properties of monodisperse chitosan-bound Fe3O4 magnetic nanoparticles for removal of Cu(II) ions. J Colloid Interface Sci 2005;283:446–51. https://doi.org/10.1016/J.JCIS.2004.09.010.

[18] Lopez JA, González F, Bonilla FA, Zambrano G, Gómez ME. Synthesis and characterization of Fe3O4 magnetic nanofluid. Revista Latinoamericana de Metalurgia y Materiales 2010;30:60–6.
